# Obesity-Associated Cancers: Evidence from Studies in Mouse Models

**DOI:** 10.3390/cells11091472

**Published:** 2022-04-27

**Authors:** Ho Lee

**Affiliations:** 1 Division of Cancer Biology, Research Institute, National Cancer Center, Goyang 10408, Korea; ho25lee@ncc.re.kr; Tel.: +82-31-920-2274; Fax: +82-31-920-2279; 2 Graduate School of Cancer Science and Policy, National Cancer Center, Goyang 10408, Korea

**Keywords:** obesity, obesity-associated cancer, mouse cancer model

## Abstract

Obesity, one of the major problems in modern human society, is correlated with various diseases, including type 2 diabetes mellitus (T2DM). In particular, epidemiological and experimental evidence indicates that obesity is closely linked to at least 13 different types of cancer. The mechanisms that potentially explain the link between obesity and cancer include hyperactivation of the IGF pathway, metabolic dysregulation, dysfunctional angiogenesis, chronic inflammation, and interaction between pro-inflammatory cytokines, endocrine hormones, and adipokines. However, how the largely uniform morbidity of obesity leads to different types of cancer still needs to be investigated. To study the link between obesity and cancer, researchers have commonly used preclinical animal models, particularly mouse models. These models include monogenic models of obesity (e.g., *ob/ob* and *db/db* mice) and genetically modified mouse models of human cancers (e.g., *Kras*-driven pancreatic cancer, *Apc*-mutated colorectal cancer, and Her2/neu-overexpressing breast cancer). The experimental results obtained using these mouse models revealed strong evidence of a link between obesity and cancer and suggested their underlying mechanisms.

## 1. Introduction

Obesity has become prevalent worldwide, with its rate doubling in the last 50 years. One-third of people worldwide are estimated to be obese by 2025, according to the World Obesity Federation [1,2,3]. Adult males and females are overweight and obese if their body mass indices (BMIs) are 25–30 and >30, respectively. Obesity has received considerable attention because it is a predominant contributor to insulin resistance, type 2 diabetes mellitus (T2DM), heart disease, stroke, and liver disease, causing metabolic, biomechanical, and psychosocial health problems in our societies.

Obesity involves a state of excess fat or the abnormal accumulation of fat throughout the body, mainly white adipose tissue [4,5]. Adipose tissue is the primary storage site of excessive energy derived from food intake. It is also well known as an endocrine organ because it secrets numerous peptide hormones and cytokines, adipokines that cause pro-atherogenic and pro-inflammatory states. There are two different depots of white adipose tissue in humans: visceral adipose tissue (VAT) and subcutaneous adipose tissue (SAT) [6,7]. Over the years, the molecular, cellular, and anatomical differences between VAT and SAT have become well known. VAT is mainly located in the mesentery and omentum, and SAT is located under the skin. VAT has more inflammatory and immune cells, a diminished capacity for differentiating preadipocytes, and a greater portion of large adipocytes than SAT. Visceral adipocytes are more metabolically active, sensitive to lipolysis, and more insulin-resistant than SAT adipocytes. Whereas peripheral obesity resulting from an accumulation of SAT is not associated with a high risk of human pathologies, abdominal obesity resulting from an accumulation of VAT has been associated with an increased risk of human diseases such as type 2 diabetes and hypertension.

Numerous complex environmental factors could be the main drivers of the current obesity and diabetes crisis [1,2,3]. Environmental factors include an obesogenic environment associated with the ready availability of inexpensive, high-calorie food and declining physical activity. Genetic factors could also be the cause of obesity. Although a high proportion of people are exposed to an environment promoting the development of obesity and related diabetes, they remain lean because of genetic factors, indicating that there are innate genetic factors affecting our susceptibility to the development of obesity and diabetes. In addition, this genetic-factor-related predisposition to obesity and diabetes involves an interaction between several genetic risk factors. Research on the interaction among these environmental and genetic risk factors could reveal these diseases’ environmental and genetic predispositions. These investigations on the causes of obesity and related diseases are crucial for developing new strategies to prevent, ameliorate, or even reverse the diseases’ detrimental and injurious effects.

Recently, many researchers have paid considerable attention to the intimate relationship between obesity and cancer, called ‘adiponcosis’ [3,8,9,10,11]. Numerous epidemiological studies have revealed that obesity is closely associated with a high risk of more than 13 different types of cancer, including those of stomach, colon, liver, kidney, pancreatic, ovarian, endometrial, postmenopausal breast, and prostate. Excess adiposity causes an increase in lipid intermediates, increased leptin levels, impaired insulin signaling, insulin resistance, and increased levels of circulating IGFs. In addition, excess adiposity causes decreased circulating adiponectin which has a critical role in reducing free fatty acids, improving lipid profiles, and decreasing inflammatory cytokines. These metabolic dysregulations are closely linked to tumorigenesis due to increased cell proliferation and migration, angiogenesis, and decreased cell death. Insulin promotes tumorigenesis directly or indirectly through reduced levels of circulating IGF-binding protein which results in increased levels of IGF-1 and IGF-2 [12]. In addition, the insulin signaling pathway promotes cancer cell survival and proliferation via the RAS/RAF/MAPK kinase/ERK pathway. Epidemiological studies revealed an association between increased levels of insulin and cancer risk, including breast, pancreatic, and endometrial cancers, indicating that hyperinsulinemia may be a key risk factor for cancer in patients with obesity and diabetes. Moreover, pro-inflammatory immune cells and hypertrophied adipocytes aggravate the inflamed microenvironment. Recently, it has been reported that high-fat diet (HFD)-induced obesity functionally impairs CD8^+^ T cells in the murine tumor microenvironment, accelerating tumor growth [13]. These results indicate that metabolic changes in the cells of overweight or obese individuals could also contribute to cancer, metastasis, and chemotherapy resistance.

However, how the largely uniform morbidity of obesity leads to different types of cancer has not been examined [3]. In addition, although there is strong evidence of a link between obesity and tumorigenesis, the underlying mechanism remains elusive because isolation of obesity from its associated abnormal manifestation is difficult and because there is a lack of suitable preclinical animal models that, similar to human patients, spontaneously develop obesity-linked cancer. Therefore, suitable preclinical models are indispensable [14,15,16]. To achieve these research goals, researchers have developed and used the majority of laboratory animals available, including non-mammalian species (zebrafish and nematodes), rodents (mice and rats), large animals (dogs and pigs), and non-human primate models. Research with rodents, especially mice, has been at the forefront of scientific advances in the study of obesity and related diseases. For example, mouse and rat models have played an essential role in identifying leptin and ghrelin genes, which are crucial due to their major roles in appetite, body weight, and energy balance. In addition, researchers have generated various types of genetically engineered mouse models (GEMMs) that spontaneously develop cancers and can therefore be considered surrogate models of human cancers [17,18].

This review highlights studies that have used mouse models to elucidate the relationship between obesity and cancer and describe their usefulness in the related research. I first introduce and describe commonly used obesity models, followed by mouse models that are regarded as surrogate models of human cancers, especially genetically engineered models. Finally, among the approximately 13 cancer types highly correlated with obesity, I focused on three types of human cancers—pancreatic, colon, and breast cancers—because these are known to be the best genetic and histopathological models for human cancers.

## 2. Pros and Cons of Mouse Models in Biomedical Research

Mice have numerous advantages over other animal models, such as worms, flies, *Xenopus*, and zebrafish, because they have similar immune, endocrine, nervous, cardiovascular, and skeletal systems to humans [16,19]. The genome of the mouse has a high similarity to that of humans (85% of the protein-coding region), and their small size facilitates the planning of high-throughput studies, making them a cost-effective model. In addition, mice develop diseases that affect the whole body and are similar to those of humans, including cancer, neurodegenerative disease, heart disease, glaucoma, and diabetes. These mouse models of human diseases are commonly chosen for oncology, neuroscience, and other studies. For example, the first demonstration of the efficacy of immune checkpoint blockade using mouse models laid the foundation for human clinical success [20]. Their generation time is relatively short (about 10–12 weeks between birth and giving birth), which means that we can observe several generations within 1 or 2 years. Most of all, conventional genetic and molecular tools are available to manipulate their genome. These tools have generated and subsequently analyzed most genetically engineered animal models in the last 30 years, helping us understand each gene’s role in the living organism. Lastly, mice are commonly used as preclinical or co-clinical models in drug development and therapeutics [17,21,22,23]. The use of mice as surrogates of human disease allows us to see how patients might respond to drugs (candidates) and treatments before they are given to patients, which is critical in ensuring their safety.

However, mouse models still have limitations and drawbacks in biomedical studies. Mice are less reliable models of human disease because there are differences in the networks linking genes and disease between mice and humans. Mice frequently fail to mimic human disease accurately and predict drug efficacy and adverse effects in drug development and preclinical studies [17,21]. There are well-known examples of failed clinical trials that showed successful preclinical results using mouse models, including TGN1412, an anti-CD28 monoclonal antibody for treating immunological disease; IPI-926, the Hedgehog pathway antagonist for treating chondrosarcoma; and MMP inhibitors for cancer and other diseases [24]. Therefore, we should consider the evolutionary factor and the similarity between mice and humans before conducting studies using mouse models.

## 3. Polygenic and Monogenic Mouse Models for Obesity Studies

In general, obesity can be categorized into two groups, monogenic and polygenic obesity [1]. Polygenic obesity, or common obesity, is caused by numerous genetic variants, which is only a minor risk factor. In polygenic obesity, environmental factors are critical for obesity features, but genetic factors have a modest contribution. Genes that affect polygenic obesity have been identified in candidate genes studies and genome-wide association studies (GWAS). Studies on common variants in candidate genes revealed that variants in only six genes (*ADRB3*, *BDNF*, *CNR1*, *MC4R*, *PCSK1*, and *PPARG*) are strongly associated with obesity. Recently, GWAS linking common genetic variants to obesity identified about 100 candidate genes associated with obesity. For example, common variants in the intron of FTO are strongly associated with BMI. The increasing number of genetic variants associated with obesity indicates that polygenic (common) human obesity is a polygenic disease with an inter-individual heterogeneity. The most common polygenic obesity model is the C57BL/6 inbred mouse, which develops hyperphagia-induced obesity in an obesogenic condition [16,25,26]. Obesity in these models is not caused by one mutation but by errors at multiple sites within the genome.

Monogenic obesity is generally rare, severe, and has an early onset because it involves either single-gene defects or chromosomal deletions inherited in a Mendelian ratio. Representative mouse models of monogenic obesity are obese and diabetes mouse lines with homozygote mutations in *ob* and *db* genes, respectively [16,25,26].

### 3.1. Mouse Models with a Defect in the Leptin Signaling Pathway

Leptin mutations cause hyperphagia, which results in obesity [16]. Leptin functions via the leptin receptor (LEPR), a single-transmembrane domain receptor of the cytokine receptor family. There are mouse models with mutations in leptin or leptin receptor genes and/or insensitivity to the leptin response because of mutations, resulting in extreme leptin resistance. Of them, *ob/ob* and *db/db* mice are the most common (Table 1). These models mimic the manifestations of obesity and T2DM in humans. At the Jackson Laboratory, two mutant clones, *ob/ob* and *db/db* mice, were reported in 1950 and 1966, respectively [27,28]. Although *ob/ob* mice (also known as B6 ob and *Lep^ob^*) were severely obese, *db/db* mice (also known as *Lepr^db^*) were severely diabetic but moderately obese. In parabiosis experiments, *ob/ob* mice surgically joined with *db/db* mice rapidly lost body weight and appetite and developed hypoglycemia [29,30]. Normal and lean mice parabiosed to *db/db* mice showed a similar response (weight loss), but the parabiosis did not affect the *db/db* mice. These parabiosis experiments revealed that a circulating factor is essential for regulating appetite, food intake, and energy use. In addition, the phenotypic similarity of *ob/ob* and *db/db* mice indicated that the two genes affect the same signaling pathway regulating appetite. The *ob* gene was identified in 1994 by positional cloning and is now called *Lep* [31]. The *ob/Lep* gene’s uncovering led to the identification and cloning of numerous related genes with roles in the leptin signaling pathway, including *Lepr*, *Mc4r*, *Pomc*, and *Pcsk1*. Subsequent work on these genes revealed that they also affect energy intake, expenditure, and body weight control and are frequently involved in human metabolic syndrome [32,33].

The obese mutation (*ob*) is recessive, and *ob/ob* mice gain excess body weight and deposit excess fat even under restricted diet conditions [34]. Their obese phenotype appears at about four weeks of age. The mice exhibit obesity, hyperphagia, transient hyperglycemia, glucose intolerance, and increased plasma insulin. They are also subfertile and show impaired wound healing. Another *ob/ob* mouse, called BTBR obese, is marked by severe hyperglycemia, progressive insulin resistance, glucose intolerance, progressive hypertriglyceridemia, and critical features of early diabetic nephropathy and diabetic neuropathy in humans [35]. The mice also have features of diabetic retinopathy and early neuronal developmental defects such as retinal function, inner retinal thinning, and cell loss [36]. These mice were developed through introgression of the *ob* allele from B6.V-*Lep^ob^* into *BTBR T^+^ Itpr3^tf^* using marker-assisted backcrossing for six generations [37].

*db/db* mice, which have a spontaneous mutation in the *db/Lepr* gene in a C57BLKS background, exhibit many features, including an uncontrolled increase in blood sugar, severe depletion of pancreatic beta cells, and death by ten months of age [38,39]. The mice also show peripheral neuropathy, myocardial disease, delayed wound healing, and subfertility.

**Table 1:** Mutant and transgenic mice of the leptin signaling pathway.

Name	Mutations or Transgene	Cancer Phenotype	Obesity Phenotypes	Others
*ob/ob*	Mutation of the *ob/Lep* gene [31]	Enhanced PDAC progression in KOC mouse [40]	Obesity, transient hyperglycemia, glucose intolerance, increased plasmid insulin	Defect in the development of mammary ductal epithelium [41]
*db/db*	Mutation of the *db/Lepr* gene [32]	Early tumor onset and poor survival in diabetic MMTV-neu mice [42]	More diabetic than *ob/ob* mouse	
NSE-LEPR	Expression NSE-LEPR-B [43]	Not determined	Reconstitution of leptin receptor signaling in a neuron.	Restoration of ductal epithelium development in *ob/ob* or *db/db* mice [44]

### 3.2. Effect of the Genetic Background in ob/ob and db/db Mice

The genetic background has a significant effect on the phenotypes and manifestations of obesity and diabetes in *ob/ob* and *db/db* mice [16,40]. In a C57BL/6 background, both mice manifest morbid obesity and only transient hyperglycemia and pancreatic beta cell hypertrophy, not atrophy, but, in a C57BLKS background, manifest chronic hyperglycemia and beta cell atrophy. Therefore, they are considered models of phase I and phase II diabetes in a C57BL/6 background and phase III in a C57BLKS background. In general, *ob/ob* mice are kept in a C57BL/6 background, and *db/db* mice are in a C57BLKS background. These differences in genetic background partly endow *ob/ob* and *db/db* mice with phenotypic differences (severe obesity vs. severe diabetes, respectively).

Beyond the difference in the genetic background, *ob/ob* mice are just obese and *db/db* mice are more diabetic [45,46]. Previous studies have reported that inflammation, the microbiome, bile acid, fatty acids, and bacterial components could affect these phenotypic differences between the mice. Although recent studies have reported that the microbiome difference and novel markers of obesity and diabetes are intimately associated with dysregulated blood glucose, much remains to be resolved about why different phenotypes and manifestations occur in response to mutations in the same pathway. Indeed, investigation and understanding of the phenotypic differences associated with leptin signaling would give us new insight into and knowledge of new therapeutics to treat obesity and diabetes or their related diseases.

### 3.3. Limitations of Mouse Models of Obesity

Although these mouse models represent the phenotype of human obesity and diabetes well and are used as surrogate models of the disease, they still have some limitations and drawbacks. Diabetic patients often develop severe complications and additional afflictions due to uncontrolled blood glucose. These afflictions include nephropathy, neuropathy, steatosis, impaired wound healing, retinopathy, and response to uncontrolled blood glucose levels. Although all mouse models of obesity or diabetes have one or more diabetic complications, no single model develops all complications and severe diabetic retinopathy. Therefore, beyond the characterization of previously developed models, it is essential and critical to developing additional models that are suitable for obesity and diabetes studies.

In addition, *ob/ob* or *db/db* mutations disturb the leptin signaling pathway, resulting in a defect in the development of mammary ductal epithelium [41]. Therefore, these mice have not been considered suitable for functional studies of the role played by the *Lep* and *Lepr* genes in obesity-associated tumors, particularly breast cancer. Chua and colleagues generated transgenic mice, including the brain-specific long form of leptin receptor (NSE-LEPR-B) transgene, which reconstitutes leptin receptor signaling in neurons (Table 1) [43]. The brain-specific expression of leptin receptors completely rescued the metabolic phenotype shown in *db/db* mice and fully restored ductal epithelium development in the mice. This transgenic mouse model is used to investigate the role of peripheral leptin signaling in mammary tumorigenesis.

## 4. Selection of Mouse Models for Obesity Studies

When choosing mouse models for studying obesity, we should consider the following: the genetic nature of the phenotype, strain background, sex, environmental stimulus, degree of characterization, phenotype onset and severity, and related phenotype [16]. Mutation types and strain backgrounds should be considered when selecting the mice and the experimental design because they are crucial to the phenotype severity. Obese and diabetes phenotypes can be monogenic or polygenic: in some models, obesity is monogenic, and diabetes is polygenic. The phenotypes often depend on stimuli, such as dietary differences that can critically affect the metabolic profile and experimental results. For example, changes in the fatty acid ratio (unsaturated to saturated) and the diet’s physical form (solid vs. liquid) resulted in different experiment data [47,48,49]. The phenotypes may be more severe in one sex, generally male. In a strains, male mice show greater susceptibility to diet-induced obesity (DIO) and develop obesity early and with higher penetrance than female mice [50,51,52]. Male TALLYHO mice develop hyperglycemia and overt diabetes, but female mice do not develop hyperglycemia [53]. While male FATZO mice develop obesity in a pre-diabetic state with slow progression to overt diabetes on a normal diet, female mice develop obesity. However, female mice do not manifest any diabetic features [54].

## 5. Investigation of the Link between Obesity and Pancreatic Cancer

Epidemiological studies have revealed that pancreatic cancer is caused by obesity and that pancreatic cancer is a high-risk factor for developing diabetes [55]. The most common malignancy of the exocrine pancreas is pancreatic ductal adenocarcinoma (PDAC), which comprises over 90% of cases. Oncogenic *KRAS* mutations are the most common mutation in PDAC patients, with a frequency exceeding 90% [56]. In addition, tumor suppressors such as *CDKN2A*, *TP53*, and *SMAD4* are mutated in greater than 95%, 50–70%, and 55% of PDAC patients, respectively. Even though revolutionary cancer therapeutics have been developed, including combination chemotherapy, small molecule-mediated targeted therapy, and immunotherapies, the survival rate of PDAC patients is still low, at about 10%.

### 5.1. Kras-Driven PDAC in a Mouse Model

The KPC mouse is one of the most-studied GEMMs of PDAC and considered the most appropriate surrogate model of human PDAC in terms of clinical and histological characteristics [57] (Table 2). Hingorani and colleagues have generated the KPC model with endogenous control and concomitant expression of *Kras^G12D^* and *Trp53^R172H^* in the pancreas [58]. This concomitant expression of oncogenic *Kras* and *Trp53* shortens the time of tumor progression from pancreatic intraepithelial neoplasia (PanIN) lesions to metastatic adenocarcinoma. In the model, spontaneous cancer can cause metastasis in the lung and liver about 2.5 months after birth. Although the KPC mouse is a successful model for the study of PDAC, it still has some limitations, such as a limited impact on the progression of other diseases, such as diabetes and infrequent signs of para-neoplastic diabetes [59]. From this point of view, the KC mouse may be a more useful model in obesity studies. These transgenic animals express the oncogenic *Kras^G12D^* alleles in acinar cells of the pancreas and show preneoplastic PanIN lesions that ultimately progress to invasive and metastatic PDAC [58]. However, invasive and metastatic cancer development in the mice is relatively slow, with PanIN-3 lesions detected at 4–6 months and full-blown metastatic cancer after 12 months of age. Another GEMM of PDAC, the KPP mouse, expresses the *Kras^G12D^* oncogenes and involves the loss of a functional *Pten* gene in the pancreas [60]. KPP mice show a progressive loss of skeletal and adipose mass due to pancreatic tumors and also display a similar gene ontology in the muscle to cachectic patients. Therefore, KPPC mice are regarded as a useful model in the preclinical study of cachexia. Recently, Collins and colleagues developed a new mouse model of PDAC, the iKras model (*Ptf1a*-Cre;LSL-rtTA;LSL-*Trp53^R172H^*;TetO-*Kras^G12D^*) [61]. In this mouse, *Kras^G12D^* is inducible, which leads to reversible and pancreas-specific expression of the oncogenic *Kras* gene. Compared with the KPC model, iKras model mice have a mean survival of about seven months, more rapid formation of PDAC, and metastasis with a larger tumor mass.

### 5.2. Study of Obesity Using Kras-Driven PDAC Models

**Figure 1:**
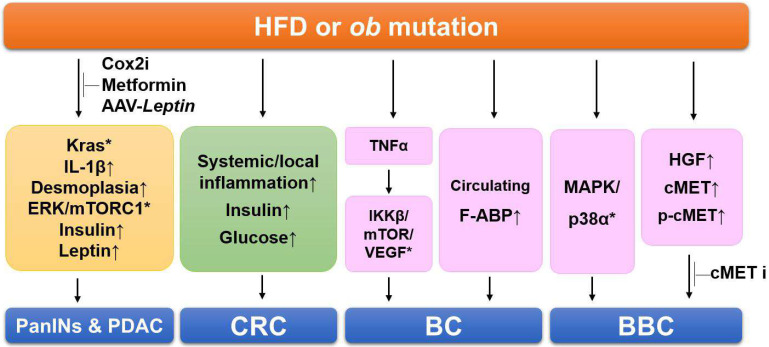
Summary of the signaling pathways involving obesity-linked cancers in mouse models. HFDs cause Kras activation, inflammation, fibrosis, and increased insulin and leptin levels, resulting in PDAC progression. Administration of Cox2 inhibitor (Cox2i), metformin, and AAV-*Leptin* prevents HDF-induced and manifestations in PDAC models. HDFs increase systemic and local inflammation, elevate insulin and glucose levels in the colorectal cancer (CRC) models. In the breast cancer (BC) models, obesity increases several genes related to proliferation and the serum levels of A-FABP. HFDs activate multiples kinases of MAPK/p38 and increase hepatocyte growth factor (HGF) secretion in basal-like breast cancer (BBC) models. Administration of cMET inhibitor (cMETi) prevents tumor development in BBC-bearing mice. Upward-pointing arrows, increased levels of indicated proteins/adipokines; *, activation of indicated signaling pathways.

Several studies using these *Kras*-driven PDAC models reported that a HFD promotes the development of pancreatic cancer. Accordingly, subsequent studies aimed at identifying the relationship between obesity and pancreatic cancer and the specific mechanisms involved are ongoing. Analysis of KC mice subjected to a HFD revealed that chronic inflammation and autophagy dysregulation play a role in diet-induced pancreatic cancer progression and genetic alteration, contributing to mortality [75]. *Ob/ob* mice have been crossed with KC mice to model obesity-associated pancreatic cancer, generating KCO mice. KCO mice are obese and show an increased primary ductal tumor burden, enhanced PDAC progression, and a dramatically shortened lifespan compared with non-obese KC mice [40]. These phenotypes were more severe than those in HFD models. This experiment also revealed that obesity promotes tumorigenesis independent of new driver mutations, such as *Trp53*, *Cdkn2a/p16*, and *Smad4* mutations, frequent in PDAC. To verify whether the inflammation induced by a HFD and the accompanying upregulation of cyclooxygenase-2 (Cox2) increase PDAC progression, KC mice were crossed with *Cox2* conditional knockout mice, generating *Cox2^flox/flox^*;LSL-*Kras^G12D^*;*Ela*-CreERT [76]. A HFD increases Kras activity, fibrotic stroma, the numbers of PanIN lesions, and PDAC incidence (Figure 1). Despite the dramatic increase in the number of PanIN lesions, inflammation, and fibrosis in LSL-*Kras^G12D^*;*Ela*-CreERT mice fed a HFD, there was no evidence for an increase in these phenotypes in *Cox2^flox/flox^*;LSL-*Kras^G12D^*;*Ela*-CreERT mice. In addition, the administration of Cox2 inhibitor to KC mice prevented the tumorigenic effects of the HFD. These results indicate that HFDs can activate oncogenic *Kras* via Cox2, causing inflammation and fibrosis in the pancreas and tumor development.

Desmoplasia is a key feature in pancreatic cancer associated with accelerated tumor growth and the impaired delivery of anti-cancer drugs due to reduced perfusion [56]. Obesity is known to promote inflammation and fibrosis in the normal pancreas. An experiment with KPC and iKras mice revealed that desmoplasia is promoted by a HFD in the models [62]. Adipocyte-secreted IL-1β recruits tumor-associated neutrophils, which activate pancreatic stellate cells. Consistent with these observations, depletion of tumor-associated neutrophils, inhibition of IL-1β, or inactivation of pancreatic stellate cells prevents the rapid obesity-mediated progression of PDAC. These results suggest that aggravation of desmoplasia is a critical mechanism of obesity-promoted PDAC progression and that clinically available anti-fibrotic or inflammatory agents could be used to treat PDAC in obese patients.

To investigate whether anti-diabetic drugs affect PDAC development, *Kras*-driven cancer models have been subjected to a HFD and/or metformin, a widely used anti-diabetic drug [77]. Metformin administration prevented HDF-induced manifestations, including increased body weight, hepatic steatosis, depleted intact acini, PanIN lesion formation, and activation of ERK and mTORC1 in the pancreas (Figure 1). Metformin also normalized the HFD-induced hyperinsulinemia and hyperleptinemia and completely abrogated the HFD-induced further increase in PDAC incidence. Based on these results, an anti-diabetic drug could be a novel chemopreventive drug for PDAC.

To verify the effect of weight loss on tumorigenesis, KCO mice, including an *ob* mutation, have been chosen because the obesity phenotype can be rapidly reversed through leptin restoration [40]. An adeno-associated virus-*Leptin* (AAV-*Leptin*) leads to sustained leptin secretion in mice through a single intramuscular injection. Multiple phenotypes of leptin deficiency, including obesity, hyperglycemia, and subfertility, can be reversed by AAV-*Leptin* administration (Figure 1). Interestingly, AAV-*Leptin* administration before advanced tumor development delays tumor progression proportional to the degree of body weight loss. However, AAV-*Leptin* administration after advanced tumor development causes loss of body weight without affecting tumorigenesis and survival. The study showed that islet cholecystokinin is aberrantly expressed in response to obesity, promoting oncogenic *Kras*-driven pancreatic ductal tumorigenesis. These results suggest that an obesity-associated microenvironment change drives PDAC progression and implicates endocrine–exocrine signaling beyond insulin in PDAC development.

## 6. Investigation of the Link between Obesity and Colorectal Cancer (CRC)

Experimental results indicate that obesity is a critical risk factor for colon cancer [78] and that colon cancer risk can be reduced by preventing or reversing obesity through a calorie-restricted diet. Investigation and identification of these associations’ molecular mechanisms will help to reveal novel therapeutic targets and develop approaches for treating obesity-related CRC. For this purpose, several mouse models of colon cancer have been used in the context of obesity, including *Apc* mutant mice and the azoxymethane (AOM)-treated models [65,66,79,80,81].

### 6.1. Mouse Models of CRC

Carcinogen-induced mouse models of CRC were first developed more than 80 years ago. Since then, considerable numbers of animal models have been generated to investigate tumorigenesis in the colon [63,64,82]. GEMMs of intestinal/colon cancer have the same mutation found in human patients and show similar pathogeneses to sporadic and inherited human colon cancers, dependent on the activation or inactivation of specific molecular pathways (Table 2). One of the earliest GEMMs of colon cancer is the *Apc^Min^* mouse (Min = multiple intestinal neoplasias), which was developed in 1990 in the laboratory of William Dove [83] and has been widely used for over 30 years in various fields. The *Apc^Min^* mouse includes a germline mutation in the *Apc* gene, an autosomal dominant loss-of-function *Apc* gene caused by a stop codon at codon 850 generated by exposure to N-ethyl-N-nitrosourea (ENU), a highly potent carcinogen. In each animal, heterozygous *Apc^Min^* mice develop more than 100 intestinal polyps, mainly located in the small intestine. The mutation most frequently found in human colon cancer patients is that in adenomatous polyposis coli (APC); this mutation is observed in over 80% of cases. Therefore, *Apc^Min^* mice have commonly been used as the model of the human familial adenomatous polyposis (FAP) syndrome. Several additional models with *Apc* mutations have been generated in subsequent work, most of which have truncating mutations in the gene [63,64]. The *Apc^∆716^* mouse was generated by inserting a neomycin cassette into Apc codon 716, generating a truncated peptide (~80 kDa). This mutation causes many adenomas, mainly in the small intestine, and a reduced number in the large intestine and fewer extra-intestinal lesions, similar to *Apc^Min^* mouse. The *Apc^1638N^* mouse was generated by inserting a neomycin cassette into Apc codon 1638. These animals develop 5–6 intestinal adenomas per animal and exhibit a broad spectrum of extra-intestinal manifestations, including cutaneous cysts and multifocal desmoids. Although *Apc^1638N^* mice develop intestinal tumors, the mice are less tumorigenic, have a longer latency than *Apc^Min^* mice, and develop invasive adenocarcinoma, which is rarely found in *Apc^Min^*, as well as splenomegaly and desmoid formation. Given these characteristics, the mice are considered to resemble human CRC more closely. AOM is an alkylating agent that generates free radicals. Administration of AOM to C57BL/6 mice causes mutations in β-catenin and tumor formation with low incidence [84]. AOM treatment of *Apc^Min^* mice increases the incidence of colon cancer and the number of polyps, but tumor formation predominantly occurs in the small intestine

### 6.2. Investigation of the Link between Obesity and Colon Cancer

The number of polyps increases and the spontaneous development of multiple intestinal neoplasias in *Apc^Min^* mice is accelerated by a HFD [65,79]. In addition to an increase in intestinal polyp formation in the *Apc^Min^* mice, exposure to a HFD increases systemic and local inflammation before the onset of obesity, as well as metabolic syndrome-associated characteristics, including an elevated level of insulin or glucose [65,66] (Figure 1). AOM-induced CRC models have also examined if diet-induced obesity affects colon cancer progression [80,81]. In the experiment, male C57BL/6J mice fed regular chow or a HFD for eight weeks were administered AOM to cause colon cancers. The mice were classified into four groups: regular chow, HFD, regular chow switched to a HFD, and a HFD switched to regular chow. The results indicated that colon cancer development is promoted by prior HFD-induced obesity, even without weight gain and HFD maintenance, meaning that colon cancer can be promoted by obesity itself, without the additional effects of a HFD or genetic alteration.

Mouse cancer models have also tested the effects of dietary components on obesity-related cancer. For example, walnuts mitigate cancer risk and include various bioactive components with anti-inflammatory and antioxidant effects. Therefore, walnuts have the potential to protect or counteract pathways that initiate or drive the development of obesity-related tumors. Using C57BL/6J mice, *Apc^1638N^*, *Apc*^Δ*14*^, and MC38 colon cancer cells, researchers have investigated the effects of walnuts on intestinal homeostasis and tumor growth or attenuation in the context of HFDs and obesity [67]. The intake of walnuts dramatically protects against intestinal tumor growth and progression and preserves the function of intestinal stem cells in the context of HFDs and obesity. These results are additional evidence that some diets (e.g., walnuts) have the potential to break the obesity–colon cancer link.

Obesogenic conditions during the intrauterine and nursing periods could affect obesity and CRC. It has been examined whether obesogenic conditions in various life periods influence obesity and intestinal tumorigenesis in adult *Apc^Min^* mice [85,86]. The results found that exposure to an obesogenic condition in utero and during the nursing period (45% fat) caused an increase in body weight in both control and *Apc^Min^* mice and increased the number of intestinal polyps in *Apc^Min^* offspring as adults compared with a control diet (10% fat). These results indicate that obesogenic conditions in the intrauterine and nursing periods could be critical for susceptibility to dietary fat-induced obesity and intestinal tumor development.

## 7. Investigation of the Link between Obesity and Breast Cancer

Obesity is associated with an approximately 40% increased risk of breast cancer recurrence and death, resulting in poor survival outcomes [87]. In particular, obesity strongly correlates with breast cancer risk in postmenopausal women [88,89] and is found in about 50% of all breast cancer cases in older women [90]. This correlation is established in estrogen receptor-positive breast cancer but is less well established in human epidermal growth factor receptor 2-positive and triple-negative subtypes. Although several mechanisms have been proposed for the link between obesity and cancer, the detailed and established molecular mechanisms of obesity-linked breast tumorigenesis remain elusive.

### 7.1. Mouse Models of Breast Cancer

To generate GEMMs of breast cancer, several oncogenes have been targeted in the mammary gland, such as Myc, PyMT, Her2/neu, and Hras, using mouse mammary tumor virus long terminal repeat (MMTV-LTR) or whey acidic protein (Wap) promoters [91,92,93,94,95]. Based on molecular profiling of mammary tumors from mouse models of breast cancer, individual tumors were clustered together on a dendrogram, suggesting that some models (MMTV-neu, MMTV-PyMT, Wap-Myc, and Wap-Int3) showed similar features to the luminal subtype of human breast cancer and that other models (C3(1)-Tag, Wap-Tag, and *Brca1*-deficient models) were similar to the basal subtype (Table 2).

MMTV-PyMT mice express polyomavirus middle T antigen driven by MMTV-LTR, restricting its expression to the mammary epithelium. This mouse model shows a high penetrance of early-onset mammary cancer than other mammary tumor models. In particular, palpable mammary tumors developed in MMTV-PyMT females could metastasize to the lung. MMTV-neu mice express the activated rat neu gene via the MMTV-LRT promoter. In the activated rat neu oncogene, a valine is replaced by glutamic acid at amino acid 664. Tumors formed in the mice are multifocal and stochastic, and adenocarcinoma is usually detected in older mice (about 23 weeks after birth). Based on the gene expression profile and the high expression of XBP1, a human luminal subtype-defining gene, the model is considered to represent the human luminal subtype [91,96]. MMTV-TGFα transgenic mice express human TGFα cDNA under the control of the MMTV-LTR [97,98]. The transgenic mice show abnormal phenotypes, including lobular hyperplasia, cystic hyperplasia, adenoma, and adenocarcinoma in the mammary epithelium. In addition, the mice show slow development of breast cancer, with onset at 12 months after birth, representing a useful model of postmenopausal human breast cancer. MMTV-Wnt1 transgenic mice express Wnt1 driven by MMTV-LTR, developing breast cancer that shows luminal and basal subtype features [99]. The transgenic construct of C3(1)-Tag mice includes the simian virus 40 early-region transforming sequences driven by the regulatory element of the rat prostate steroid-binding protein C3(1) gene. Most transgenic mice (~95%) show features of the human basal-like subtype, such as high proliferation and high expression of keratins 5 and 17 and P-cadherin. The female mice usually develop mammary intraepithelial neoplasia similar to ductal carcinoma in situ (DCIS) by three months after birth and subsequently develop mammary adenocarcinoma by six months of age with 100% penetrance. In addition, about 10–15% of female mice develop lung metastasis.

### 7.2. Investigation of the Link between Obesity and Breast Cancer

For determining the effects of obesity on the initiation and progression of breast cancer, two mouse models of obesity have been used: one is an orthotopic mammary tumor-bearing genetic model (*ob/ob* mice), while the other is an orthotopic mammary tumor-bearing diet-induced obesity model [100]. The results showed that the initiation and development of breast cancer were promoted by obesity in the models and that the expression was increased of several genes related to proliferation, including *TNFa*, *VEGF*, *IKKβ*, and *mTOR*, indicating that TNFα may activate the IKKβ/mTOR/VEGF signaling pathway in the tumors of obese mice (Figure 1). Other groups have also reported that the initiation of breast cancer and lung metastasis was increased by a HFD containing soybean oil in MMTV-PyMT mice [68,69]. Furthermore, they identified the upregulation of pro-inflammatory cytokines, adipokines, and angiogenic factors, which were closely associated with the increased and aggressive features of mammary tumors induced by the HFD. From these studies, a molecular interpretation may explain why the incidence of breast cancer is higher and the prognosis is worse in obese women.

To investigate whether a dietary intervention that reduced adiposity before tumor onset would reverse HFD-induced breast cancer, breast cancer models (C3(1)-Tag mice) were fed a low-fat diet (LFD) or HFD and then an obese group exposed to a HFD was switched to a LFD to cause weight loss [73,74]. Weight loss before tumor initiation inhibited the increase in HFD-induced cancer, which reduced tumor latency and preneoplastic lesions, including atypical ductal hyperplasia and DCIS. Kinome analysis explained that multiple kinases upstream of MAPK/p38α were activated by the HFD-induced weight gain and reversed by the weight loss (Figure 1). These may be novel targets in obesity-associated breast cancer, particularly basal-like breast cancer (BBC). Finally, these results suggested that tumor initiation can be promoted by the HFD-exposed microenvironment, which was reprogrammed by weight loss and the restoration of a lean phenotype. Another study of C3(1)-Tag mice determined that obesity significantly increases the secretion of hepatocyte growth factor (HGF) and decreases the latency of breast cancer [101] (Figure 1). The expression of HGF, cMET, and phospho-cMET in the normal mammary glands was elevated, accelerating BBC tumor progression compared with lean controls. In addition, weight loss significantly reversed the HFD-induced effects on latency and the activation of HGF/cMET signaling in normal mammary tissue and cMET in normal mammary and breast cancer tissue. Because obesity leads to a critical elevation in HGF/cMET secretion in breast cancer, researchers have investigated whether a small-molecule cMET inhibitor (e.g., crizotinib) has effects on BBC tumor progression in LFD- and HFD-fed C3(1)-Tag BBC mice [102]. cMET inhibitor was administered before tumor development or at an early stage. It significantly decreased tumor volume by 27.96% and 37.29% and average cancer vascularity by 35.04% and 33.52% in the LFD- and HFD-fed C3(1)-Tag mice, respectively. These results suggest that tumor development and microvascular density in basal-like tumor-bearing mice could be inhibited by cMET inhibition with crizotinib.

Recently, more and more studies have reported that metabolic changes are strongly associated with the development of breast cancer. For example, the serum levels of A-FABP are dramatically increased in obesity [98,103], which is associated with the stemness and aggressive development of breast and ovarian cancer (Figure 1). From these results, circulating A-FABP could be a new link between obesity and breast cancer and a new potential therapeutic target to treat obesity-associated tumors.

The effect of obesity in tumorigenesis was examined in female MMTV-PyMT ovary intact and ovariectomized mice [104]. Obesity caused an increase in inflammation and hormone production, resulting in an increase in tumorigenesis in premenopausal MMTV-PyMT mice. However, a HFD had no significant effect on tumorigenesis in a postmenopausal MMTV-PyMT. Other experiments with MMTV-Wnt1 cancer cell line revealed that calorie restriction and rapamycin inhibit tumor growth in postmenopausal models [72] (Table 2). In the experiment, MMTV-Wnt1 mammary tumor cells were transplanted to ovariectomized C57BL/6 mice.

The NSE-LEPR-B transgenic mouse has been used to investigate the role of peripheral leptin signaling in breast cancer progression; this transgenic mouse was bred to a MMTV-PyMT mammary tumor mouse model [44]. In double transgenic mice, the microenvironment displays high local levels of leptin under obese conditions, which increases the leptin receptor-mediated pathway, including elevated PI3K, ERK1/2, and STAT3 activation, resulting in the acceleration of tumor progression. Experiments using MMTV-Wnt1 cancer cell lines also revealed the critical of leptin in breast cancer development [105]. Leptin deficiency caused reduced growth and tumor-initiating activity of transplanted tumors. These results from the experiment provide strong in vivo evidence that peripheral leptin exerts a novel local paracrine function within the mammary gland and that leptin plays a major role in breast cancer development.

## 8. Conclusions

This review introduces monogenic obesity models and GEMMs commonly used in studies linking obesity and cancer. Additionally, recent studies using mouse models to investigate the link between obesity and cancer and its mechanisms are summarized. These mouse models made a decisive contribution to elucidating the obesity genes. As a result, many obesity studies were undertaken, resulting in a significant amount of information on obesity and related diseases. Obesity studies using cancer model mice also investigated the effects of obesity on cancer progression and demonstrated the efficacy of various anti-dietary drugs and signaling pathway inhibitors.

Although there have been many advances in obesity research using the current mouse model to determine its relationship with cancer, efforts to solve the remaining questions will continue. Developing appropriate mouse models for this purpose is an essential objective. In addition, the development of more diverse models of human obesity and cancer for obesity and cancer research will promote the elucidation of new targets and the identification of new therapeutic agents.

## Figures and Tables

**Table 2:** Mouse models commonly used in studies on obesity-cancer.

Name	Mutations or Transgenes	Obesity Phenotypes	Cancer or Disease Phenotypes
KPC	*Kras^G12D^*;*Trp53^R172H^*; *Pdx1*-Cre [58]	HDFs promote desmoplasia [62]	Limited impact on the progression of other diseases, such as diabetes, due to rapid tumor progression
KC	*Kras^G12D^*; *Pdx1*-Cre [58]	More useful model in obesity studies than KPC mouse	Slow progression in invasive and metastatic cancer
KPP	*Kras^G12D^*; *Pten(f/f)*;*Ptf1a*-CreERT [60]	Preclinical model of cachexia	Displaying a similar gene ontology in muscle to cachectic patients
*APC^Min^*	Stop codon at codon 850 [63,64]	HFDs increase systemic and local inflammation before the onset of obesity [65,66]	Developing more than 100 intestinal polyps, but rare invasive adenocarcinoma
*Apc^1638N^*	Insertion of the neomycin cassette in codon 1638 [63,64]	HFDs accelerate tumor development and increased multiplicity [67]	Close resemblance to human CRC
MMTV-PyMT		HDFs enhance primary tumorigenesis and metastasis [68,69]	Similar features with the luminal subtype of human breast cancer
MMTV-neu		HFDs effect, controversial [70,71]	Similar features with the luminal subtype of human breast cancer
MMTV-TGFα		HFDs increase adiposity and shorten mammary tumor latency	Useful model of postmenopausal human breast cancer.
MMTV-Wnt1		CR * inhibit transplanted tumors in postmenopausal model [72]	Features of the luminal and basal types.
C3(1)-Tag		HFDs increase tumor initiation [73,74]	Similar features with the basal subtype of human breast cancer

* CR, calorie restriction.

## Data Availability

Not applicable.

## Abbreviations

Type 2 diabetes mellitus, T2DM; visceral adipose tissue, VAT; subcutaneous adipose tissue, SAT; high-fat diet, HFD; genetically engineered mouse model, GEMM; genome-wide association studies, GWAS; leptin receptor, LEPR; colorectal cancer, CRC; azoxymethane, AOM; diet-induced obesity, DIO; pancreatic ductal adenocarcinoma, PDAC; pancreatic intraepithelial neoplasia, PanIN; multiple intestinal neoplasia, Min; ductal carcinoma in situ, DCIS; mouse mammary tumor virus long terminal repeat, MMTV-LTR; whey acidic protein, Wap; low-fat diet, LFD; basal-like breast cancer, BBC; hepatocyte growth factor, HGF.
